# All’s Not Well(ens) that Ends Well: Case Study of Significant Thebesian Veins Mimicking Wellens Syndrome

**DOI:** 10.51894/001c.12881

**Published:** 2020-06-08

**Authors:** Keith Frank

**Affiliations:** 1 Cardiology Garden City Hospital

**Keywords:** wellens syndrome, acute coronary syndrome, thebesian vein

## Abstract

Thebeisan veins are microfistulous connections between a coronary arterial branch directly to a ventricular or atrial chamber. Extensive thebesian veins that empty into the left ventricle can cause typical chest pain symptoms, troponin elevation, and ischemic EKG changes from coronary steal leading to acute coronary syndrome in extreme cases. Literature review exposed a consistent pattern of EKG findings among patients with extensive thebesian veins involving all three major coronary arteries. We present a case study as an example of this rare anatomic finding of extensive thebesian veins draining into the left ventricle causing acute coronary syndrome in a symptomatic patient with elevated troponin and ischemic changes on EKG. This same EKG pattern that is present in our patient was discovered to be consistent among available case studies reviewed that had included an EKG tracing in their report. A newly proposed association between the ischemic changes on EKG due to extensive thebeisan veins and those of a severe proximal left anterior descending coronary artery stenotic lesion was discovered. The newly discovered consistency in the EKG pattern with acute coronary syndrome caused by extensive thebesian veins is the same pattern as that seen in Wellens Syndrome.

## INTRODUCTION

A coronary artery fistula is an abnormal luminal connection (i.e., communication via a tubular structure) between a coronary artery and either a vein, another artery, or even a ventricular chamber.^1^ Thebeisan veins are microfistulous connections between a coronary artery directly to a ventricular or atrial chamber.^2,3^ They are important during congenital cardiac circulation, but generally have little contribution to myocardial circulation in adults.^2,3^ When thebesian veins persist into adulthood, they are typically asymptomatic and drain into the right heart. However, they have been known to drain into the left heart as well.^4^

When a significant number of microfistulae exist, left-to-left shunting occurs, meaning blood that should be continuing downstream in the arterial system has taken an alternative pathway creating a new circuit of blood flow that doubles back on itself. In this case, blood that should be conducted from the aorta to coronary arteries and coronary capillaries then to the coronary sinus and right atrium has found an abnormal alternative pathway.

This alternative pathway involves blood flowing from the aorta to the coronary arteries back into the left ventricle (LV) and then the aorta again, creating a loop. Thus, blood circulation is diverted from a left sided structure (the coronary arteries), back into another left sided structure (the LV) and does not pass though the finer myocardial capillary network nor the pulmonary circulation.

These extra pathways of blood flow can typically cause patients to experience anginal symptoms, dyspnea (i.e., difficulty breathing), exertional limitations, abnormal stress testing results, diastolic volume overload, and even myocardial ischemia with possible Acute Coronary Syndrome (ACS) (i.e., myocardial infarction or ischemia).^4^ In the case of extensive thebesian veins, ACS is due to coronary steal (i.e., alteration in circulatory patterns) causing ischemia to the myocardium.^5,6^

To have a single coronary artery in communication with a cardiac chamber is rare (0.08% - 0.3%),^5^ but having all three major coronary arteries empty into a single cardiac chamber with enough flow to elicit coronary steal is extremely rare. In a healthy patient, the total contribution to aortic flow from thebesian veins is 0.12% - 0.43%.^7^ The author has found no empirical studies available to quantify the occurrence of this cardiac abnormality.

There are multiple ways for clinicians to discover a significant thebesian venous system, one of which is through coronary arteriography.^4^ Without surgical options to date, treatment options center around medical therapy consisting of beta blocker antihypertensives and possible nitrate vasodilitators.^5^ There exists some controversy regarding the benefit of nitrate therapy in these patients due to the possible risk of exacerbating the coronary steal phenomenon, although no consensus has been reached.^2^ Treating comorbidities and other coronary artery disease (CAD) risk factors would be appropriate as well.

## CASE REPORT

**Figure 1:**
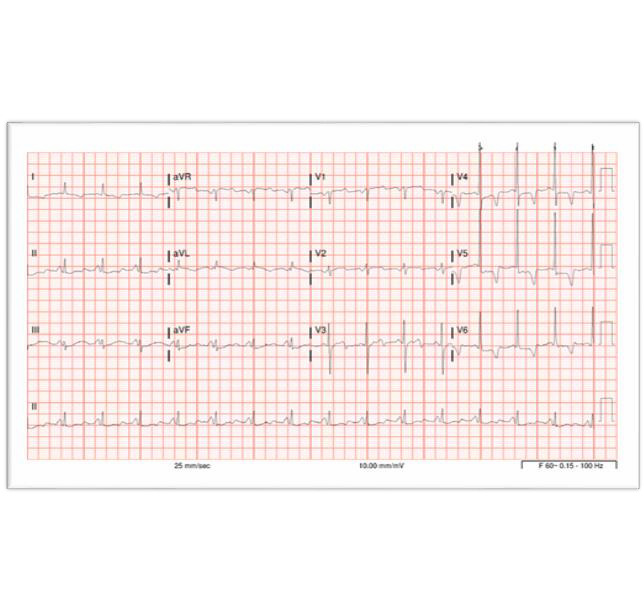
EKG on admission showing normal sinus rhythm with biphasic T waves in V3, T wave inversions in I, aVL, V4 – V6, and ST depressions in V4 – V6 and aVF.

Our patient was a black female in her early 60’s who presented to the emergency room (ER) secondary to two days of progressive substernal chest pressure with associated mild shortness of breath. Her past medical history included Type 2 diabetes mellitus, hypertension, tobacco abuse, and stroke. This patient’s physical exam was negative for any significant findings. Her electrocardiogram (EKG) was concerning for ACS. (Figure 1) Troponin I level was elevated at 1.05 ng/ml (normal range being <0.04 ng/ml). A prior myocardial perfusion test performed 12 months ago failed to identify any reversible or fixed cardiac defects.

The patient was admitted with a diagnosis of ACS non-ST segment elevated myocardial infarction in which the EKG did not display ST segment elevations (as seen in most cases of acute myocardial infarction), but labs were consistent with myocardial injury (elevated troponin level). Her physicians were concerned that the culprit artery causing ischemic disease was the Left Anterior Descending (LAD) based on her EKG findings meeting criteria for Wellens Syndrome. She additionally reported currently undergoing an extremely stressful economic situation, which raised concern for possible Takotsubo Cardiomyopathy (i.e., stress induced cardiomyopathy) in which there is no coronary artery disease, but severe left ventricular myocardial dysfunction that is present with EKG changes mimicking ACS.

**Figure 2:**
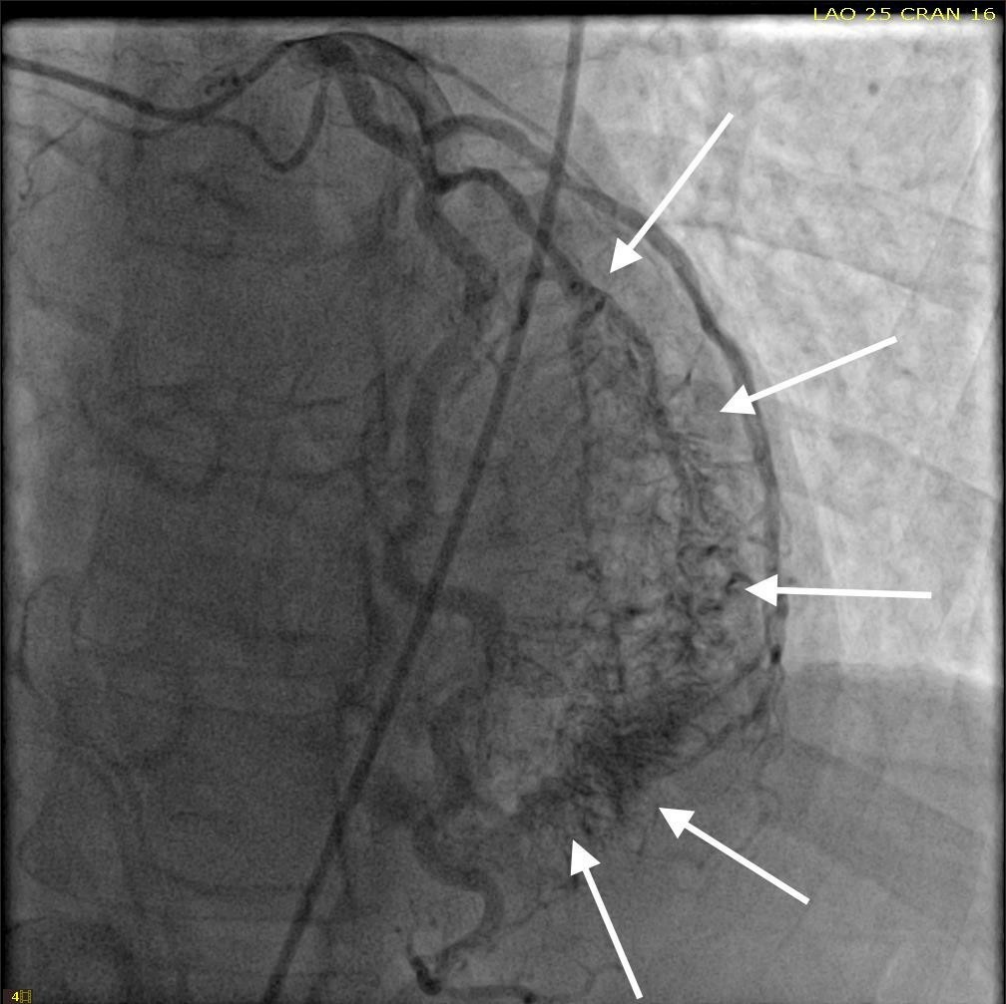
Left coronary artery angiography showing a significant thebesian vein network, as indicated by the solid arrows, involving the LAD, LCx, and their associated branches

**Figure 3:**
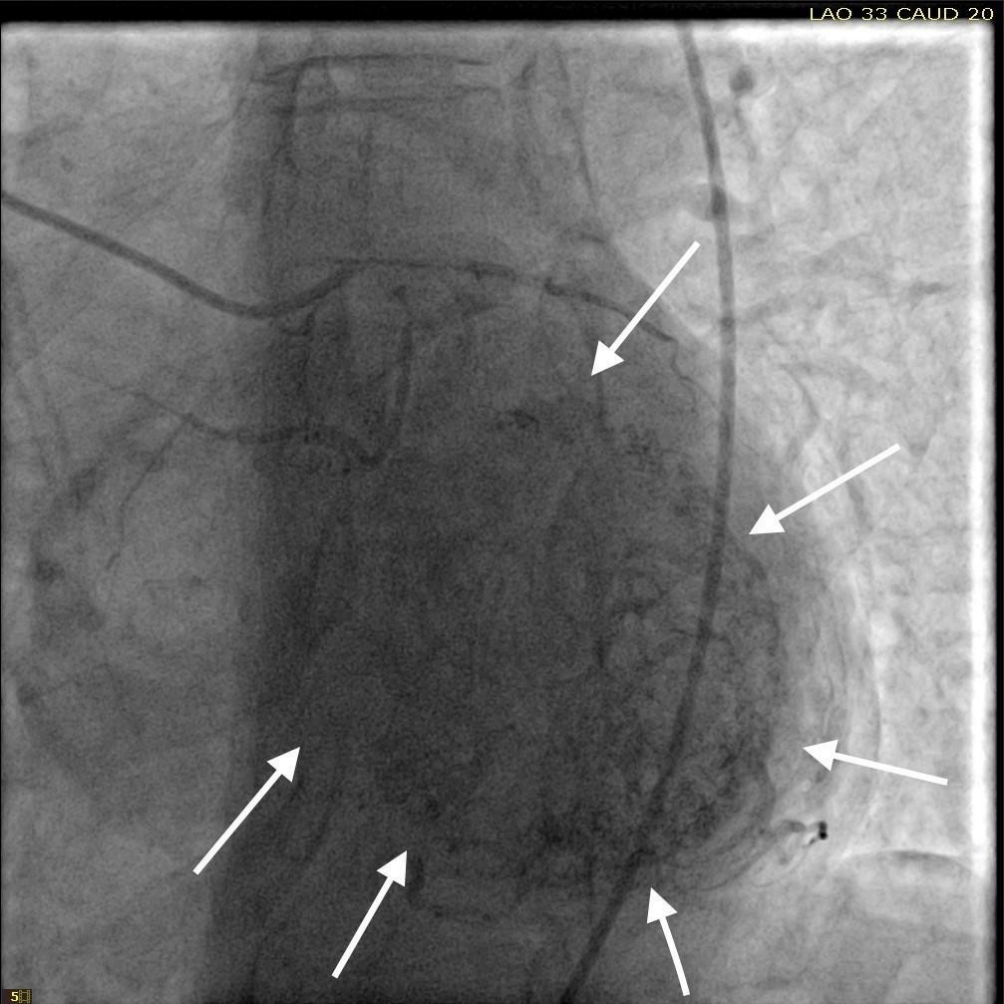
LV end diastole after left coronary angiography showing filling of the LV chamber (outlined by the solid arrows) with intravenous contrast via thebesian veins from the LAD, LCx, and their associated branches.

**Figure 4:**
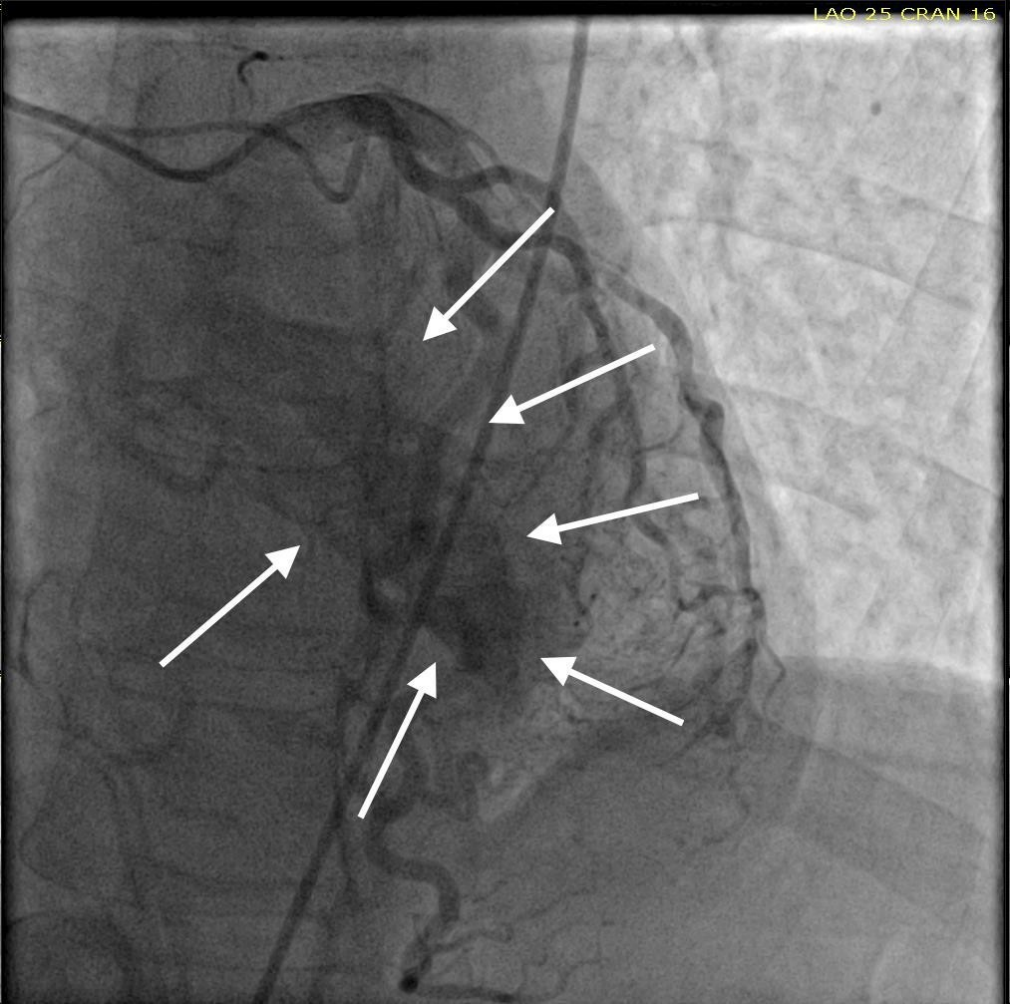
LV end systole after left coronary angiography showing filling of the LV chamber (outlined by the solid arrows) with intravenous contrast.

She was taken for a diagnostic left heart catheterization which showed mild to moderate disease in multiple coronary arteries with severe stenosis of a small caliber mid right sided Posterior Descending Artery (rPDA) not amenable to percutaneous intervention. The cardiac catheterization also revealed an extensive network of microfistulae forming communications between all three major coronary arterial branches (i.e., LAD, Right Coronary Artery [RCA], and Left Circumflex [LCx]) and the LV cavity (Figures 2, 3, and 4). This patient’s flow through her microfistulous communications was so brisk that a perfectly interpretable left ventriculogram was visualized with coronary angiographic imaging via a hand injection of contrast into the left coronary arterial system (Figures 3 and 4).

It was not possible to quantitate this patient’s amount of flow through her extensive thebesian venous network since it is generally extremely technically difficult to do so.^7^ However, it was obvious that this woman’s total thebesian venous flow far exceeded the upper limit of what is considered typical since her LV ejection fraction (normal being in the range of 54 - 74% in women) could easily be interpreted with a hand injection during left coronary artery angiography. On the second day after admission, complete 2D echocardiography was performed only showing moderate LV hypertrophy.

Despite the known severe stenotic lesion of the rPDA, this patient’s EKG did not correlate with the macrovascular disease found on angiography. The microfistulae were then suspected of being the cause of ACS due to coronary steal phenomenon.^5,6^

This patient was treated with metoprolol tartrate (i.e., antihypertensive beta blocker used for anti-anginal properties), isosorbide mononitrate (i.e., a nitrate used for anti-anginal properties), clopidogrel (adenosine diphosphate P2Y_12_ receptor inhibitor used to treat coronary artery disease by inhibiting normal platelet function), and low dose aspirin (81-100mg once per day). No intervention was performed on the stenotic coronary arterial segments since they were small caliber vessels not amenable to stenting. The patient’s symptoms resolved prior to hospital discharge, and on the third day she was stable for discharge home. She was advised to follow up with Cardiology as an outpatient for continued care. This would entail optimizing her CAD medication regimen according to current guidelines, minimizing her CAD risk factors, and monitoring to ensuring she has no symptom recurrence.

## DISCUSSION

In related case reports, significant coronary artery to LV microfistulae have been observed in patients with and without symptoms of ACS.^1,2,6,8,9^ In these published cases, similar EKG changes of T wave inversions in at least one precordial lead as well as ST depressions were imaged.^1,2,6,8,9^ Some patients, as with the patient in this case report, presented with elevated troponin levels^2,6^ and some were within normal limits^1,8^ in spite of their ACS symptoms.

Left heart catheterization was performed on each of these earlier patients which led to the unveiling of the extensive thebesian venous network draining into the LV. One of these case studies involved an asymptomatic patient with significant thebesian vein abnormalities and an EKG demonstrating the same pattern consistent with other patients diagnosed with symptomatic thebesian veins.^9^ These cases, in combination with the case presented here, support the conclusion that extensive hemodynamically significant thebesian veins can cause EKG changes, symptoms, and signs of ACS.

Importantly, the author’s review of cases with obtainable EKGs did show a consistent pattern of T wave inversions in precordial leads, with some EKGs also having negative T waves in inferior leads and/or ST depressions as well.^1,2,5,8^ More specifically, a biphasic T wave in V3 appeared unanimously in all patients with extensive thebesian veins, with their EKG showing an appearance consistent for a diagnosis of Wellens Syndrome.^10,11^

The author’s conclusion is that hemodynamically significant thebesian veins can cause the same ischemic EKG changes similar to those seen in Wellens Syndrome, and that extensive thebesian veins (or microfistulae) should be added and considered as a differential diagnosis when proximal LAD ischemia is suspected from a Wellens Syndrome EKG pattern. This type of ischemia to myocardial tissue can be due to the coronary steal phenomenon and mimic a severe proximal LAD stenotic lesion and a Wellens Syndrome EKG pattern.

The most striking similarity between the found literature and the case presented here is the biphasic T wave seen in V3, as previously mentioned. All patients, except for one, also had some significant signs or symptoms of myocardial ischemia. This single case study that involved an asymptomatic patient with the EKG pattern described demonstrated a significant thebesian venous networked despite the absence of symptoms.^9^ It is difficult to fully compare differences between this patient’s case with other published cases without the films, pictures, or full operative report from the left heart catheterizations. It is also difficult for the author to draw complete clinical conclusions from such a small patient population with accuracy.

## CONCLUSIONS

This evidence should raise awareness for an extensive thebesian vein network being: 1) a possible mimicker of Wellens Syndrome, 2) capable of causing hemodynamically significant alterations in myocardial perfusion, 3) a cause of myocardial ischemia, and 4) the etiology of significant anginal signs and symptoms. Further research, including specific treatments for the clinical course of this condition, need to be considered to more fully evaluate optimal care approaches for these patients.

### Conflict of Interest

The authors declare no conflict of interest.
